# Supplementary figures and images for: Breviscapine alleviates podocyte injury by inhibiting NF-κB/NLRP3-mediated pyroptosis in diabetic nephropathy
Source: PeerJ. 2023 Feb 13;11:e14826. doi: 10.7717/peerj.14826 (PMC9933739; doi:10.7717/peerj.14826)

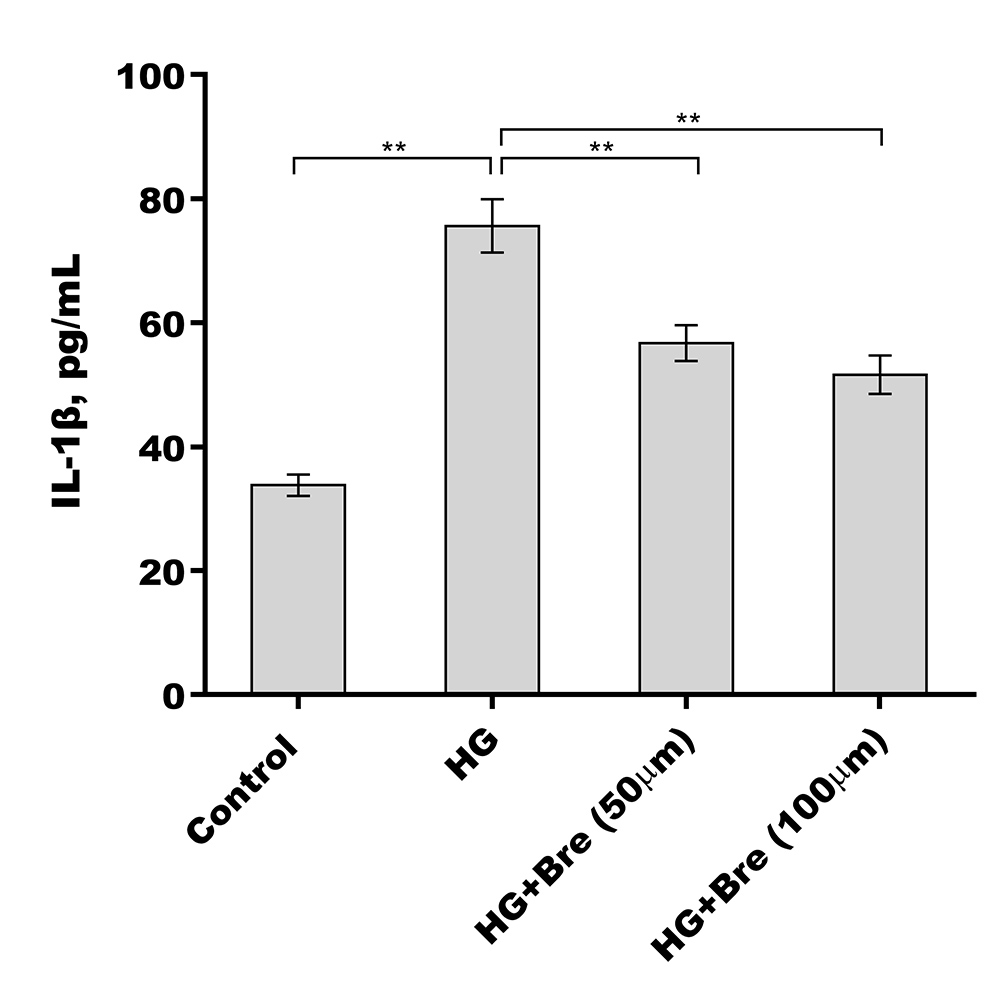

Supplement: Supplemental Information 1 [file peerj-11-14826-s001.png]
