# Supplementary material for: Breviscapine alleviates podocyte injury by inhibiting NF-κB/NLRP3-mediated pyroptosis in diabetic nephropathy
Source: PeerJ. 2023 Feb 13;11:e14826. doi: 10.7717/peerj.14826 (PMC9933739; doi:10.7717/peerj.14826)
Supplement: Supplemental Information 2 [file peerj-11-14826-s002.docx]

**Supplementary Table S1. qRT-PCR primes used in the study.**

α-SMA（Acta2）

Forward primer GCTACGAACTGCCTGACGG

Reverse primer GCTGTTATAGGTGGTTTCGTGGA

Podocin

Forward primer CACCGCTGCATTGAGAATGG

Reverse primer GACCTGCTACTTCTCCCGTG

Synaptopodin

Forward primer CGGGGCTTAGTGAGGAAGATG

Reverse primer CCCTGATGTGGCGTCTAAGC

IL-18

Forward primer CCTTTGAGGCATCCAGGACAA

Reverse primer ACAGCCAGTGTTCAGTCAGC

IL-1β

Forward primer TGCCACCTTTTGACAGTGATG

Reverse primer ATGTGCTGCTGCGAGATTTG

β-actin

Forward primer GGAGTACGATGAGTCCGGC

Reverse primer GTGTAAAACGCAGCTCAGTAACA
